# Maximum swimming speeds of sailfish and three other large marine predatory fish species based on muscle contraction time and stride length: a myth revisited

**DOI:** 10.1242/bio.019919

**Published:** 2016-08-19

**Authors:** Morten B. S. Svendsen, Paolo Domenici, Stefano Marras, Jens Krause, Kevin M. Boswell, Ivan Rodriguez-Pinto, Alexander D. M. Wilson, Ralf H. J. M. Kurvers, Paul E. Viblanc, Jean S. Finger, John F. Steffensen

**Affiliations:** 1 Department of Biology, Marine Biological Section, University of Copenhagen Strandpromenaden 5, Helsingør DK-3000, Denmark; 2 IAMC-CNR, Istituto per l'Ambiente Marino Costiero, Consiglio Nazionale delle Ricerche, Località Sa Mardini, Torregrande, Oristano 09170, Italy; 3 Leibniz-Institute of Freshwater Ecology and Inland Fisheries, Müggelseedamm 310, Berlin 12587, Germany; 4 Faculty of Life Sciences, Humboldt-Universität zu Berlin, Invalidenstrasse 42, Berlin 10115, Germany; 5 Department of Biological Science, Marine Sciences Program, Florida International University, North Miami, FL 33181, USA; 6 School of Life and Environmental Sciences, University of Sydney, Heydon-Laurence Building A08, Sydney New South Wales 2006, Australia; 7 Max Planck Institute for Human Development, Center for Adaptive Rationality Lentzeallee 94, Berlin 14195, Germany; 8 Bimini Biological Field Station Foundation, 9300 SW 99st, Miami, FL 33176, USA

**Keywords:** Muscle twitch, Maximum swimming speed, *Istiophorus platypterus*, *Sphyraena barracuda*, *Euthynnus alletteratus*, *Coryphaena hippurus*

## Abstract

Billfishes are considered to be among the fastest swimmers in the oceans. Previous studies have estimated maximum speed of sailfish and black marlin at around 35 m s^−1^ but theoretical work on cavitation predicts that such extreme speed is unlikely. Here we investigated maximum speed of sailfish, and three other large marine pelagic predatory fish species, by measuring the twitch contraction time of anaerobic swimming muscle. The highest estimated maximum swimming speeds were found in sailfish (8.3±1.4 m s^−1^), followed by barracuda (6.2±1.0 m s^−1^), little tunny (5.6±0.2 m s^−1^) and dorado (4.0±0.9 m s^−1^); although size-corrected performance was highest in little tunny and lowest in sailfish. Contrary to previously reported estimates, our results suggest that sailfish are incapable of exceeding swimming speeds of 10-15 m s^−1^, which corresponds to the speed at which cavitation is predicted to occur, with destructive consequences for fin tissues.

## Abstract (precis)

**Summary:** Using muscle contraction measurements, this work provides evidence that sailfish are most likely unable to reach the extremely high speeds claimed by previous research and popular articles.

## INTRODUCTION

Animal maximum speeds play a significant ecological role, particularly in the context of predator-prey interactions (Domenici, 2001; Wardle, 1975; Wilson et al., 2013). Early estimates of maximum swimming speeds suggest that some large predatory fishes such as sailfish (*Istiophorus platypterus*) and black marlin (*Makaira indica*) may reach values exceeding 30 m s^−1^ (Barsukov, 1960; Lane, 1941). More recently, work on billfishes using data storage tags has shown that blue marlin (*Makaira nigricans*) rarely exceed speeds of 2 m s^−1^, with a maximum of 2.25 m s^−1^ (Block et al*.*, 1992) and a study of sailfish hunting schooling sardines reported an upper speed limit of 8.19 m s^−1^, based on high-speed video and accelerometry (Marras et al*.*, 2015). However, no previous study has unequivocally tested the maximum speed attainable in billfishes. High-frequency accelerometers and high-speed video are usually confined to relatively short time intervals, reducing the chance of detection of high speed events, which are thought to occur only rarely during an animal's lifetime (Block et al., 1992; Wilson et al., 2013).

A powerful approach for estimating the maximum swimming speed potentially attainable by fish is to measure their minimum muscle contraction times (Wardle, 1975). This method capitalizes on the fact that, theoretically, fish swimming speeds are physiologically limited by the tail-beat frequency attainable in a given environment (Bainbridge, 1958; Brill, 1996; Brill and Dizon, 1979; Wardle, 1975; Wardle and He, 1988; Wardle and Videler, 1980). Estimates based on minimum muscle contraction times thus yield the theoretical maximum values attainable by fish, although the physical environment may impose further limits, as suggested by hydrodynamic models (Iosilevskii and Weihs, 2008). These models suggest that swimming animals have an upper maximum speed based on the constraints that the water imposes through either (i) power limitation on the propulsive forces needed to reach a certain swimming speed, or (ii) for swimming animals longer than ∼1 m, the speed at which cavitation occurs, with destructive consequences for the tissues (Iosilevskii and Weihs, 2008).

The goal of this study was to estimate the maximum swimming speeds potentially attainable (i.e. based on measurements of muscle contraction times) by sailfish and other large marine predators. Specifically, we aimed to test firstly whether the maximum attainable speeds by sailfish are as high as those claimed by early studies (Barsukov, 1960; Lane, 1941), and secondly, how sailfish speeds compare to other large marine predatory fish.

## RESULTS AND DISCUSSION

**Fig. 1.**
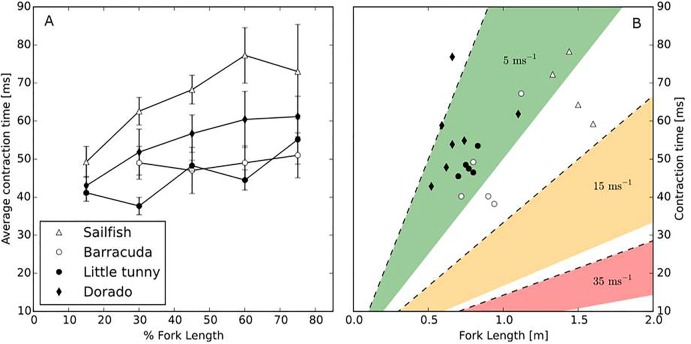
**Contraction time vs. length in four large marine predators.** (A) The average minimum twitch contraction times of the muscle (±s.e.m.) along the body of 4 different species of fish (sailfish *n*=4, barracuda *n*=5, little tunny *n*=5, dorado *n*=7). The x-axis is percentage of total length; note that the barracuda only has 4 stimulation locations due to the comparatively large head. (B) The average contraction time at 45% *L_f_* (y-axis) as a function of fork length (x-axis) for all fish. Shaded areas represent contraction times corresponding to 5, 15, and 35 ms respectively. Upper boundaries of these areas (broken lines) correspond to a stride length of 1 *L_f_* whereas the lower boundaries of the areas represent a stride length of 0.5 *L_f_*. This range (0.5-1 *L_f_*) is typical in swimming teleosts (Videler, 1993).

**Fig. 2.**
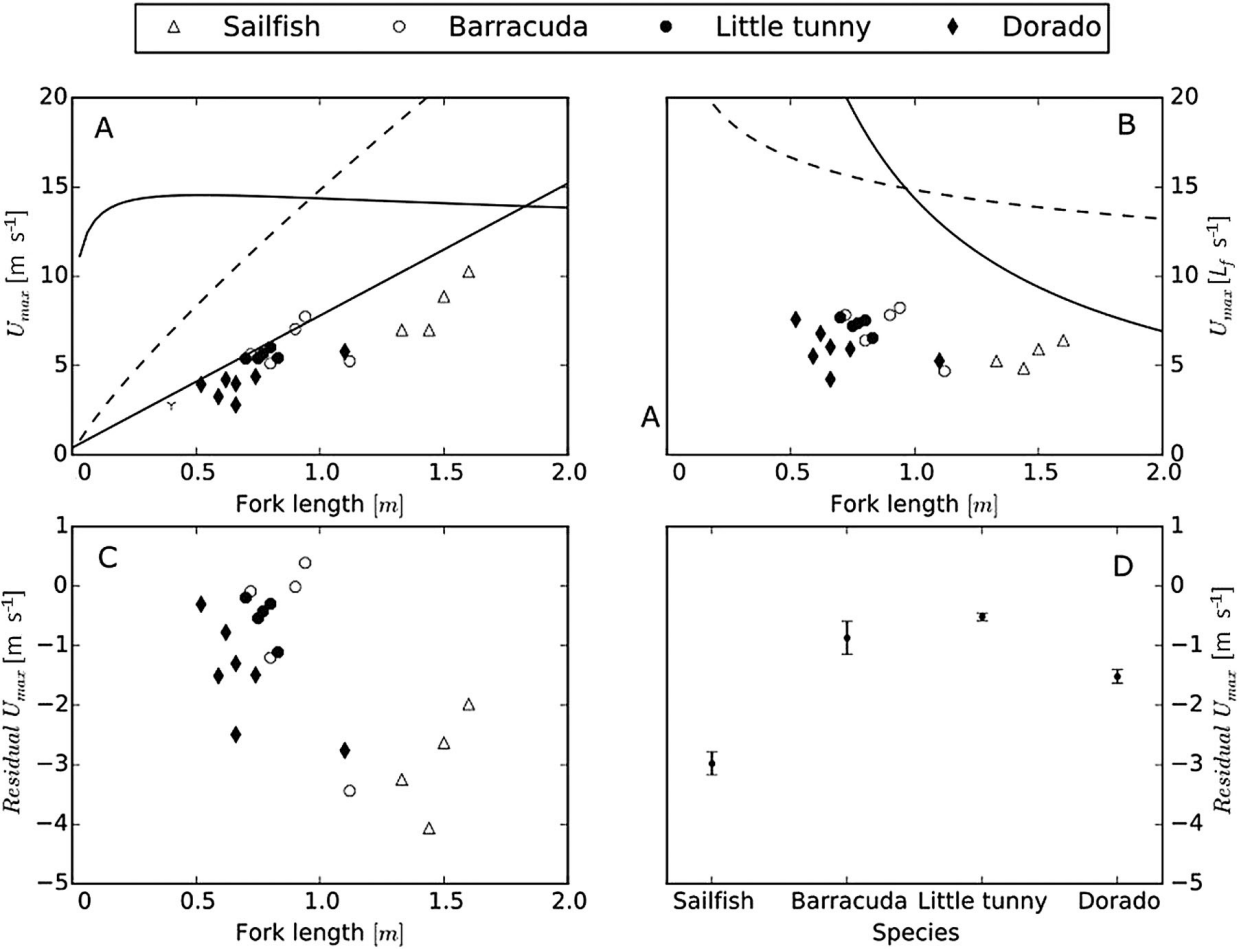
**Estimated swimming performance in four species of large marine predators.** (A,B) The calculated maximum attainable swimming speeds for the four species expressed in m s^−1^ (A) and in *L_f_* s^−1^ (B). The broken curves represent the power limitation calculated from Iosilevskii and Weihs (2008) (150W kg^−1^) and the full curves represent an estimate of maximum swimming speed caused by the cavitation limit at shallow depth with a condition factor corresponding to that of bonito (Jin et al*.*, 2015). The full line (in A) corresponds to the equation for maximal swimming speed (in m s^−1^) given by Videler (1993) (*U*=0.4+7.4 *L_f_*). (C) Residuals [deviation from expected *U_max_* based on Videler (1993)] as a function of *L_f_*. (D) Residuals [deviation from expected *U_max_* based on Videler (1993)] for each species, mean (±s.e.m.).

**Fig. 3.**
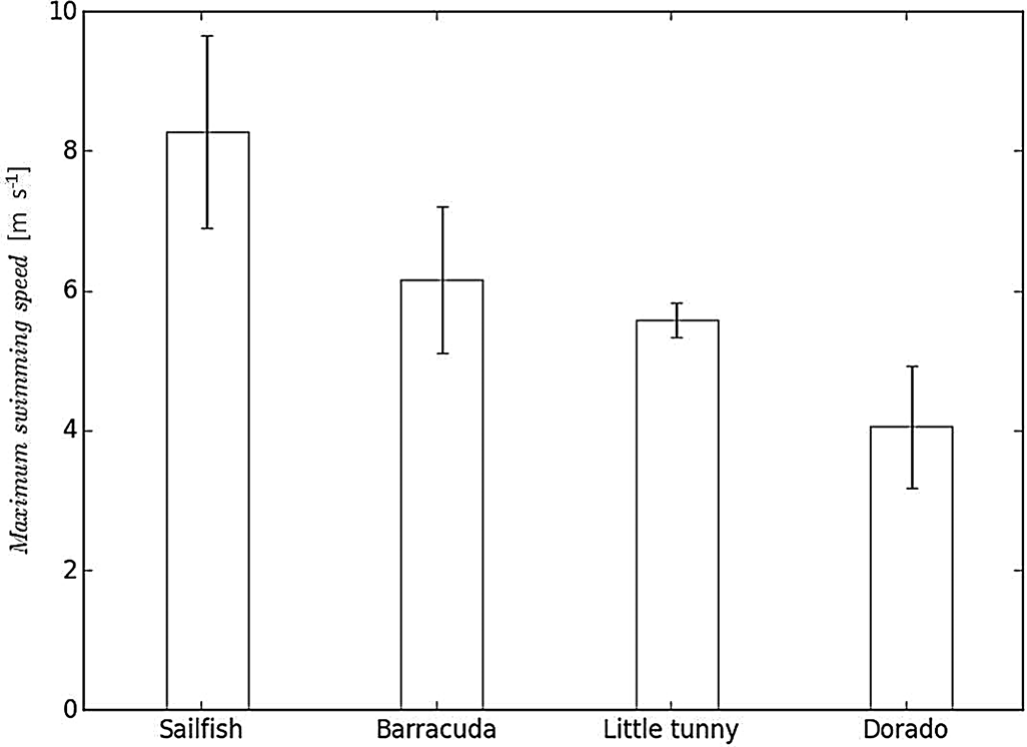
**Maximum swimming speeds (mean±s.d.) in four large marine predators.** When comparing absolute swimming speed of sailfish with that of three other large marine predators, sailfish appears to be the fastest. However, when corrected for size, the barracuda and little tunny are the top performers. (For length corrected comparison see Fig. 2D and text.)

Sea surface temperature measured at our fishing location (21° 13′ 50″ N, 86° 37′ 40″ W) was 26.6±0.7°C. The body temperature at the stimulus location [15, 30, 45, 60, 75% along the fish fork length (L_*f*_) with 0% representing the tip of the head and 100% the fork of the tail] was: sailfish: 26.8±1.1°C; barracuda: 27.6±0.5°C; little tunny: 28.9±0.8°C; and dorado: 27.5±0.6°C (one-way ANOVA: d.f.=3, *F*=10.001, *P*<0.001). Only little tunny differed significantly from the other species (*post hoc* Tukey test; *P*<0.05). Comparing the minimum twitch contraction times of the muscle along the length of the fish, we observed a general increase in contraction time from head to tail (Fig. 1A). The minimum contraction times at the 45% (*L_f_*) position were: sailfish, 68.5±7.2 ms; barracuda, 47.0±10.8 ms; little tunny, 48.3±2.8 ms; and dorado, 56.7±10.3 ms (Fig. 1B). These correspond to the following tail beat frequencies: sailfish, 7.4±0.8 Hz; barracuda, 11.1±2.1 Hz; little tunny, 10.4±0.6 Hz; and dorado, 9.1±1.5 Hz. The resulting assessments of maximum swimming speeds using contraction times from the 45% *L_f_* position in m s^−1^ (and in *L_f_* s^−1^) are: sailfish, 8.3±1.4 m s^−1^ (5.6±0.6 *L_f_* s^−1^); barracuda, 6.2±1.0 m s^−1^ (7.0±1.3 *L_f_* s^−1^); little tunny, 5.6±0.2 m s^−1^ (7.3± 0.4 *L_f_* s^−1^); and dorado, 4.0±0.9 m s^−1^ (5.4±1.0 *L_f_* s^−1^) (one-way ANOVA: d.f.=3, *F*=5.371, *P*=0.008) (Figs 2A, 3). Sailfish had significantly higher maximum swimming speeds (m s^−1^) than the other species (*post hoc* Tukey test, *P*<0.05); however, when considering size-corrected performance (i.e. residuals), sailfish had the lowest values (Fig. 2C,D; one-way ANOVA: d.f.=3, *F*=5.120, *P*=0.01). The only significant *post hoc* comparisons were those between sailfish and little tunny (*P*=0.009), and between sailfish and barracuda (*P*=0.028). Using the fastest contraction times, regardless of position on the body, higher measures for maximum swimming speed can be calculated [sailfish, 11.45 m s^−1^ (15% *L_f_*); barracuda, 6.2 m s^−1^ (45% *L_f_*); little tunny, 7.28 m s^−1^ (30% *L_f_*); and dorado, 5.2 m s^−1^ (15% *L_f_*)], on average 1.25 times faster swimming speed.

Our data suggest that sailfish are not able to achieve the extremely high speeds claimed by earlier studies (Barsukov, 1960; Lane, 1941). These speed assessments (approximately 35 m s^−1^) are based on fishermen’s records of hooked specimens and are most likely overestimations. Our estimated maximum speeds of sailfish (8.3±1.4 m s^−1^) are slightly higher than those observed during predator-prey interactions, i.e. average 7 m s^−1^ (Marras et al., 2015). This is expected because these estimates are based on unloaded muscle, i.e. fish are taken out of the water and do not consider additional effects of drag. Our estimates provide theoretical maxima; in water, fish muscles are loaded and so lower speeds are more likely. Thus, an important assumption to note when using this method is that minimum contraction time is considered independent of load. Even considering this, our estimates are still much lower than previous high estimates. Our estimates of maximum swimming speed of little tunny (5.6±0.2 m s^−1^ at a body temperature of approximately 29°C) match previously reported values of 6-8 m s^−1^ estimated using a similar method (Brill and Dizon, 1979) and video recordings (maximum speed 6.9 m s^−1^) (Yuen, 1966) on a similar species, *Katsowonus pelamis*. A tuna larger than the one we measured (i.e. 0.77 m), will be capable of higher speeds. Estimates using a similar muscle contraction time method found maximum speed of 15 m s^−1^ in *Thunnus thynnus* (Wardle et al., 1989), which is lower than the speed estimated based on rod and reel in another tuna species (i.e. 20.7 m s^−1^ in *Thunnus albacares*) (Walters and Fierstine, 1964). For barracuda, previously measured values for burst swimming speeds of approximately 12 m s^−1^ (Gero, 1952), though not as excessive as the values for billfishes (Barsukov, 1960; Lane, 1941), may also be slightly overestimated since they are approximately twice the values established here (6.2±1.0 m s^−1^).

Although it is likely that early work overestimated swimming speeds, speeds higher than those predicted based on the twitch contraction methods might theoretically be possible if fish were able to change their mode of swimming to accommodate for the otherwise lack of increase in tail beat frequency (Wardle and Videler, 1980). In order to reach speeds of 35 m s^−1^, however, sailfish would have to increase their stride length more than four times (required *L_S_*≈3.4 BL), which seems an unlikely performance because swimming fish have only rarely been observed to have a stride length exceeding 1 body length (Videler, 1993; Videler and Wardle, 1991; Wardle and Videler, 1980; Wardle et al., 1989). Therefore, unless billfishes resort to yet another mechanism to increase their speed beyond those predicted by muscle contraction measurements, the possibility that they can swim at speeds higher than 10-15 m s^−1^ is unlikely. As shown by Wardle et al. (1989), in bluefin tuna (*Thunnus thynnus*) the contraction time tends to increase towards the tail (see also Fig. 1A). This implies that at high speeds, muscles of opposite sides will progressively overlap their contractions towards the tail, stiffening the body. Previous work on other species has suggested that this might aid the transmission of the force from the anterior muscle (Altringham et al., 1993; Shadwick and Syme, 2008; Syme and Shadwick, 2002; Wardle et al., 1989).

Based on the estimated absolute speeds, sailfish appear to be the fastest of the four species investigated here, however, they were also 50-80 cm longer than the other three species and maximum speed is known to increase with fish length (Wardle, 1975). Using a length-speed relationship based on burst swimming performance of various species (Videler, 1993), we found that the size-corrected speed performance is highest in little tunny and barracuda, followed by dorado and sailfish (Fig. 2D). This agrees with the observations that sailfish do not rely on fast swimming speeds to attack their prey (i.e. observed speeds <10 m s^−1^) but rather on a stealthy approach and rapid bill movements for injuring and capturing prey (Domenici et al*.*, 2014). Although their maximum speeds are likely to be slower than previously thought, all predatory fishes investigated here have absolute maximum swimming speeds far exceeding that of their prey. Using the same length-speed relationship of (U=0.4+7.4L) suggested by Videler (1993), a 25 cm prey fish would be capable of swimming only 2.25 m s^−1^. Thus sailfish, little tunny, dorado and barracuda can swim as fast as 3.7, 2.8, 2.5, and 1.8 times the maximum speed of their prey, respectively. This implies that, based on maximum swimming speed alone, these predators would be capable of catching their prey.

The relatively high speed estimated for little tunny may partly be due to their higher muscle temperature which is known to decrease muscle contraction time (Brill and Dizon, 1979; Wardle, 1975). Little tunny showed a similar performance to barracuda, despite differing vastly in ecomorphology: little tunny is a cruising specialist, like other tuna, while barracuda's body shape and sit-and-wait lifestyle resembles another acceleration specialist, the pike (*Esox lucius*) (Webb, 1984). This suggests that elevated temperature in the little tunny may allow this species to reach a sprint speed as high as that of an acceleration specialist such as barracuda. Although elevated muscle temperature is mainly found in the red muscle (Bernal et al., 2010), a slight elevation can also be present in the white muscle of tuna (Carey and Teal, 1966). Small tuna (40-60 cm) are known to elevate their deep (red) muscle temperature by about 5°C (Block et al., 1993; Graham and Dickson, 2001), which is in line with a 2°C elevation in the deep white muscle. Dorado showed relatively poor performance, both in absolute and size-corrected speeds, which suggests that dorado rely mainly on high maneuverability (Webb and Keyes, 1981) for catching prey. It is important to note, that the assessments presented here pertain to only the reported temperatures of the fish. If we assume that sailfish can be found at 36°C then maximum swim speed of 16.6 m s^−1^ may be possible [based on a Q_10_ value of 2 (Videler, 1993, Altringham and Block, 1997)]. This speed is still much lower than the early estimates, and within the theoretical limits set by Iosilevskii and Weihs (2008).

Interestingly, none of the species investigated here showed estimated speeds higher than 10 m s^−1^. This is still below the upper limit suggested for all aquatic swimmers (Iosilevskii and Weihs, 2008). The values from Iosilevskii and Weihs (2008) are based on a model and such values have to be considered with the limitations and assumptions of the model in mind. Iosilevskii and Weihs (2008) suggest that destructive cavitation is a likely constraint on maximal swimming speeds of larger fishes, particularly at shallow depths. From a perspective of safe design, if a fish at any time exceeds their specific limits of cavitation, they would incur an increased maintenance cost (Karasov, 1986) for repairing damaged tissue. Such additional maintenance costs following excessive swimming speed would likely exceed the energy gained from the prey items caught as a result of bursting. From an evolutionary perspective, it is tempting to suggest that fish may not have evolved a muscular system capable of minimum contraction times such that they would be able to swim at speeds exceeding 10-15 m s^−1^ (depending on fish size), given that it would result in costly damage to the fins.

## MATERIALS AND METHODS

### Fish capture

All fish where caught during February 2015 in the Caribbean Sea, 5-20 nautical miles offshore the Yucatan peninsula, Cancun area (21° 13′ 50″ N, 86° 37′ 40″ W) from a sport fishing vessel. Fish were caught using rod and reel by professional fishermen, and time from initial lure strike to landing was minimized and never exceeded 15 min. Upon landing, fish were euthanized by a blow to the head by a professional fisherman, and this standard professional fishing procedure was not altered for the scientific purposes of the subsequent measurements. Experimental measurements were conducted on dead animals within 5 min from being killed.

Four fish species were collected: sailfish, *Istiophorus platypterus*, *n*=4, mean fork length (*L_f_*)±s.d=1.47±0.1 m; barracuda, *Sphyraena barracuda*, *n*=5, *L_f_*=0.89±0.1 m; little tunny, *Euthynnus alletteratus*, *n*=5, *L_f_*=0.77±0.1 m; and dorado (dolphinfish), *Coryphaena hippurus*, *n*=7, *L_f_*=0.69±0.2 m. Note that we used fork length, defined as the distance from the tip of the mouth to the tail fork, in our swimming speed calculations below. This was done to assure that measurements were comparable between species. If we would have used total length, the elongated bill of the sailfish would have been included, causing wrong estimates for fish swimming speeds. Total lengths of the different fish species were: sailfish, mean total length (*L_t_*)±s.d.=2.01±0.1 m; barracuda, *L_t_*=0.95±0.1 m; little tunny, *L_t_*=0.80±0.0 m; and dorado (Dolphinfish), *L_t_*=0.83±0.2 m.

### Measurements

Immediately after a fish was euthanized, muscle temperature was measured at 3-5 cm depth depending on fish size using a needle-K-type thermocouple (YF-160M, YU FONG, China) and muscle contraction recorded at 5 positions [15, 30, 45, 60, 75% fork length (*L_f_*)] from head to tail along the length of the fish, except in barracuda for which only the last 4 positions were used due to their elongated heads. Contraction times were measured halfway between the lateral line and the dorsal side at 2 cm depth, ensuring stimulation of the white muscle tissue. Muscle contraction was measured using a similar device as described in Wardle et al*.* (1989). The muscle was stimulated to contract via an electrical stimulus, using a Grass stimulator (Model SD9, Natus Medical Incorporated, Pleasanton, CA, USA) imposed between two hypodermic needles. Briefly, the device consisted of a bending piece of acrylic (width×length×thickness: 1.5×4.0×0.1 cm) with a hypodermic needle in either end, 2.5 cm apart, fixed using acrylic blocks of 0.5 cm thickness. The hypodermic needles were used for stimulating the muscle, and Parafilm had been added 2 cm from the tips to provide a fast assertion of penetration depth. The Parafilm (Bemis Company, Inc., Neenah, WI, USA) also limited slippage of the hypodermic needles during contraction (no slippage was observed during contraction as well). Two strain gauges glued on either side of the bending acrylic ‘bridge’ plate, placed between the hypodermic needles, allowed recording of the contraction time of the muscle. Using acrylic as the bridge is unlikely to affect the precision of the measurements, as the dimensions and stiffness of the apparatus combined allowed for a fast response time and high sensitivity to deformation. The signal from the strain gauge was via an amplifier fed to a Vernier LabPro ADDA device and acquired on a laptop with the software Vernier Logger Pro 3 (Vernier Software & Technology LLC, Beaverton, OR, USA). A minimum of eight contractions was recorded at each location on the fish and the shortest contraction time was used in the analysis. We first applied 10V to a fish, doubling this amount in case of no contraction, using a maximum of 100V (succession of stimuli: 10, 20, 40, 80, 100V), for a constant duration. Pilot tests on euthanized rainbow trout (*Oncorhynchus mykiss*) (10°C) performed at the University of Copenhagen revealed no difference in peak contraction time with increasing stimulus voltage.

### Data analysis

As maximum swimming speed (*U_max_*), we used ‘sprint speed’ spanning multiple tail beats without any acceleration. Maximum swimming speed was measured following (Wardle, 1975):
(1)
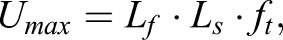
where *L_f_* is the fork length of the fish (in m), *L_s_* (proportional to *L_f_*) is the stride length (i.e. the distance that a fish moves during one complete tail beat), and *f_t_* the tail beat frequency (in s^−1^). Tail beat frequency was calculated based on the muscle contraction times (*t_c_*) (Brill, 1996; Brill and Dizon, 1979; Wardle, 1975):
(2)
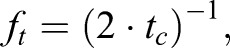
where, *t_c_* represents the time from onset of stimulus to peak contraction (Brill and Dizon, 1979; Wardle, 1975). Contraction times at the 45% *L_f_* position were used for estimating maximum speeds in figures and statistics as most muscle mass is located around 40-50% *L* (Videler, 1993), and should thus contribute the most to the propulsion of the fish. However anterior positions yield the shortest *t_c_* (Wardle et al., 1989) and therefore provide slightly faster estimates for speeds potentially attainable by a fish. For comparative reasons the mean values for these estimates have been provided in the results section.

A species-specific stride length was obtained either from our own measurements or from published values: sailfish, *L_s_*=0.76 *L_f_* observed in steady swimming during predator-prey interactions in the wild (Marras et al., 2015); barracuda, *L_s_*=0.63 *L_f_* obtained using DIDSON echograms (see below), and Boswell et al. (2008) and Mueller et al. (2010); little tunny, *L_s_*=0.65 *L_f_* based on published values of a similar species, skipjack tuna (*Euthynnus affinis*), from a swim tunnel at constant speed (Donley and Dickson, 2000); and dorado, *L_s_*=0.65 *L_f_* based on observations in a 7.3 m diameter tank for most commonly observed swim speeds (Murchison and Magnuson, 1966).

For barracuda, estimates of stride length were calculated using echograms obtained from a stationary dual frequency identification sonar (DIDSON) (Sound Metrics Corp. Bellevue, WA, USA) observing multiple barracuda steadily swimming through an ensonified area. In the initial frame of each observation, we measured the position and bearing of the fish swimming at a constant velocity with no acceleration; the position and bearing at time zero was then converted into Cartesian coordinates using the range of the center acoustic beam as a reference distance. In the subsequent frames, positional coordinates were measured, and the distance between coordinates was calculated from the difference between fish positions, corrected for changes in sonar heading and background translational motion. Time was measured using the number of frames required to perform a complete tail beat divided by the frame rate of the DIDSON sonar, which resulted in a range of 1-2.5 s of swimming time per fish. The stride length was then calculated from the positional differences over the tail beat time period as a proportion of its measured body length (Marras et al., 2015).

### Statistical analysis

We tested between species differences in temperature and absolute swimming speeds using one-way ANOVA, with *post hoc* comparison (Tukey). To compare swimming speeds across size ranges, we used a burst speed-length relationship (*U_max_*=0.4+7.4L) based on literature data on various fish species (Videler, 1993). Thus, the residuals calculated as the difference between our estimated maximum speeds and the burst speed-length relationship were compared among species using one-way ANOVA with Tukey test. Differences were considered significant at *P*<0.05.
